# A novel p38α MAPK inhibitor suppresses brain proinflammatory cytokine up-regulation and attenuates synaptic dysfunction and behavioral deficits in an Alzheimer's disease mouse model

**DOI:** 10.1186/1742-2094-4-21

**Published:** 2007-09-04

**Authors:** Lenka Munoz, Hantamalala Ralay Ranaivo, Saktimayee M Roy, Wenhui Hu, Jeffrey M Craft, Laurie K McNamara, Laura Wing Chico, Linda J Van Eldik, D Martin Watterson

**Affiliations:** 1 Center for Drug Discovery and Chemical Biology, Northwestern University, 303 E Chicago Ave, Mailcode W896, Chicago, IL 60611, USA; 2 Faculty of Pharmacy A15, University of Sydney, NSW2006, Sydney, Australia; 3 Guangzhou Institute of Biomedicine and Health, Chinese Academy of Sciences, Guangzhou Science Park, 510663, Guangzhou, China; 4 Barnes-Jewish Hospital, Washington University in St Louis, St Louis, MO 63110, USA

## Abstract

**Background:**

An accumulating body of evidence is consistent with the hypothesis that excessive or prolonged increases in proinflammatory cytokine production by activated glia is a contributor to the progression of pathophysiology that is causally linked to synaptic dysfunction and hippocampal behavior deficits in neurodegenerative diseases such as Alzheimer's disease (AD). This raises the opportunity for the development of new classes of potentially disease-modifying therapeutics. A logical candidate CNS target is p38α MAPK, a well-established drug discovery molecular target for altering proinflammatory cytokine cascades in peripheral tissue disorders. Activated p38 MAPK is seen in human AD brain tissue and in AD-relevant animal models, and cell culture studies strongly implicate p38 MAPK in the increased production of proinflammatory cytokines by glia activated with human amyloid-beta (Aβ) and other disease-relevant stressors. However, the vast majority of small molecule drugs do not have sufficient penetrance of the blood-brain barrier to allow their use as *in vivo *research tools or as therapeutics for neurodegenerative disorders. The goal of this study was to test the hypothesis that brain p38α MAPK is a potential *in vivo *target for orally bioavailable, small molecules capable of suppressing excessive cytokine production by activated glia back towards homeostasis, allowing an improvement in neurologic outcomes.

**Methods:**

A novel synthetic small molecule based on a molecular scaffold used previously was designed, synthesized, and subjected to analyses to demonstrate its potential *in vivo *bioavailability, metabolic stability, safety and brain uptake. Testing for *in vivo *efficacy used an AD-relevant mouse model.

**Results:**

A novel, CNS-penetrant, non-toxic, orally bioavailable, small molecule inhibitor of p38α MAPK (MW01-2-069A-SRM) was developed. Oral administration of the compound at a low dose (2.5 mg/kg) resulted in attenuation of excessive proinflammatory cytokine production in the hippocampus back towards normal in the animal model. Animals with attenuated cytokine production had reductions in synaptic dysfunction and hippocampus-dependent behavioral deficits.

**Conclusion:**

The p38α MAPK pathway is quantitatively important in the Aβ-induced production of proinflammatory cytokines in hippocampus, and brain p38α MAPK is a viable molecular target for future development of potential disease-modifying therapeutics in AD and related neurodegenerative disorders.

## Background

Up-regulation of proinflammatory cytokine production by activated glia has been implicated in disease progression in a variety of chronic neurodegenerative disorders, including Alzheimer's disease (AD), Parkinson's disease, multiple sclerosis, amyotrophic lateral sclerosis, and HIV-associated dementia [for selected reviews, see [1-10]]. In AD, studies with clinical samples and investigations using animal models provided strong correlations of early increases in proinflammatory cytokine levels, especially interleukin-1β (IL-1β) and tumor necrosis factor α (TNFα), prior to neurologic sequelae [5,11,12]. Causal relationships were established by demonstration of a worsening of neuropathologic outcomes as a result of experimentally manipulated increases in proinflammatory cytokines or an improvement of outcomes with treatments that decrease cytokine levels. The former includes the use of transgenic and knockout mouse models subjected to AD-relevant stress [13,14], or direct administration of cytokines to the brain [15-19]. The latter includes treatment with small molecules that suppress excessive cytokine production by glia back towards basal [20-23]. This accumulating body of evidence is the foundation of current efforts to decipher which combinations of disease-relevant stressors and signal transduction pathways might be amenable to therapeutic interventions that modulate cytokine production [for review, see [1]].

Current drugs approved for human use to modulate cytokine function are macromolecules [e.g., see [24,25]]. Although a clinical feasibility study in AD patients raises the potential of positive neurologic outcomes [26], macromolecular drugs have a number of disadvantages for clinical use in chronic CNS disorders, including high cost and inconvenient dosing regimens. Thus, there is a critical need for orally active, brain-penetrant, small molecule therapeutics that can suppress excessive proinflammatory cytokine production by glia back towards homeostasis without being pan-suppressors, such as steroids with their untoward side effects and poor ability to alter pathophysiology progression [27,28].

Recently, we developed an experimental therapeutic whose mechanism of action is reduction of excessive proinflammatory cytokine levels in the hippocampus back towards basal levels, with a resultant attenuation of synaptic dysfunction and hippocampus-dependent behavior alteration [22,23,29]. The drug, Minozac, is in clinical development. Minozac discovery and development used a *de novo *compound discovery platform interfaced with hierarchal biological screens for oral bioavailability, toxicity, brain penetrance, and stability. Compounds emerging from the platform were tested for efficacy in animal models of CNS disorders [22,23,30], employing the more unbiased functional approach to drug discovery that has proven attractive for complex disorders and initial therapy development in areas of unmet need [31,32]. Minozac, therefore, provides a precedent for selective targeting of increased proinflammatory production with positive neurologic outcomes in an AD-related neurodegenerative disease model. Minozac is not an inhibitor of p38α MAPK, an established drug discovery target for peripheral tissue diseases, such as rheumatoid arthritis, that are also characterized by increased proinflammatory cytokine production as part of disease progression [for reviews, see [33-38]]. In contrast to the extensive knowledgebase for peripheral tissue disorders, less is known about the *in vivo *contributions of the p38α MAPK signaling cascade to the brain cytokine overproduction and neurodegenerative sequelae in CNS disorders such as AD, or the potential of p38α MAPK as a therapeutic target for such disorders [for reviews, see [39,40]].

The p38 MAPK signaling cascade is activated in AD as demonstrated by staining of AD brain tissue samples for phosphorylated (activated) p38 MAPK or upstream components of the pathway [19,41-46]. Activation of the p38 MAPK pathway is also seen in AD-relevant animal models [47-51]. However, causative linkages between MAPK pathway activation and proinflammatory cytokine production by glia is mainly via cell culture studies. For example, stimulation of glial cell cultures with Aβ_1–42 _induces p38 MAPK activation [52-56] with a later induction of proinflammatory cytokines, and p38 MAPK inhibitors block the increase [see, e.g., [53,56-58]]. Therefore, there is a body of strongly suggestive evidence that brain p38α MAPK may be a viable therapeutic target for AD and related neurodegenerative disorders. Further pursuit of this hypothesis requires the use of brain-penetrant, small molecule p38α MAPK inhibitors to demonstrate restoration of Aβ-induced up-regulation of brain cytokine production back towards normal, with an associated improvement in neurologic outcomes.

In order to fill this void in knowledge and provide a foundation for future therapeutic development efforts, we employed the same chemical scaffold used for Minozac development to design and produce a novel p38α MAPK inhibitor with potential for use in studies of brain pathology alteration in AD-relevant animal models. The rationale for using chemical diversification of the Minozac scaffold is two-fold. First, analog design is one of the most successful for the development of novel small molecule drugs, with approximately two-thirds of all small molecule sales resulting from the analog approach [59]. Second, greater than 98% of small molecule drugs have inadequate blood-brain barrier penetrance [60]. Minozac [22] and the lead compound from which it was developed, MW01-5-188WH [23], use a common scaffold and have good blood-brain barrier penetrance, justifying redundant use of this scaffold in attempts to discover a p38α MAPK targeted inhibitor for altering CNS pathophysiology.

We describe here the development of a novel, orally bioavailable, brain-penetrant, non-toxic p38α MAPK inhibitor and its *in vivo *use at a low oral dose to attenuate human Aβ-induced increases in mouse hippocampus cytokine levels, consistent with the proposed mechanism of inhibitor action. Improved neurologic endpoints support the hypothesis that p38α MAPK is a viable target for future drug development.

## Methods

### Design and synthesis of MW01-2-069A-SRM

**Figure 1:**
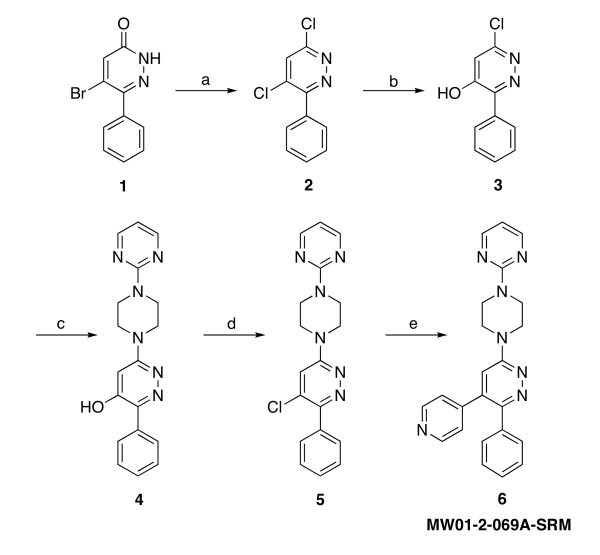
**Synthetic scheme for synthesis of MW01-2-069A-SRM**. Reactions, conditions and yields: a) POCl_3_, reflux, 2 hrs, 99%; b) NaOH, H_2_O, reflux, 2 hrs, 67%; c) 1-(2-pyrimidyl)piperazine, 1-butanol, 130°C, 26 hrs, 98%; d) POCl_3_, reflux, 3 hrs, 99%; e) 4-pyridylboronic acid, Pd(PPh_3_)_4_, K_2_CO_3_, DME, 110°C, 15 hrs, 74%.

Compound MW01-2-069A-SRM (hereafter designated 069A) was designed based on the inactive core 3-phenyl-6-(4-(pyrimidin-2-yl)piperazin-1-yl)pyridazine scaffold, which was also used in the fragment-based discovery of MW01-5-188WH and Minozac [22,23]. The scaffold was subjected to chemical diversification by introduction of the 4-pyridinyl pharmacophore as shown in Fig. 1. Pharmacophore modeling was assisted by the use of the p38α MAPK crystal structure 1YQJ in the Protein Database and the commercially available software FlexX-pharm (Tripos, Inc., St. Louis, MO). Structure-based searches of the literature to demonstrate novelty of the 069A chemical structure were done with SciFinder Scholar^® ^(American Chemical Society).

The synthetic scheme for the production of 069A is shown in Fig. 1. The synthesis of 069A was accomplished by the generation of a precursor, compound **5**, that was amenable to introduction of the pyridinyl functionality by standard cross-coupling chemistry and using commercially available reagents and catalysts (Scheme 1, step e). The generation of the precursor compound **5 **required a variation of the previously described [22,23,29] generalized synthetic scheme. This was accomplished by first (step c) introducing the amine 4-(pyrimidin-2-yl)piperazine onto the pyridazine ring to generate compound **4**, followed by a halogenation reaction to make the reactive precursor compound **5**.

Details corresponding to each step in the synthetic scheme are described below. The starting material, reagents and solvents used in this synthetic scheme are commercially available, or readily generated from commercially available materials using standard chemical reactions. The detailed reaction conditions presented for each step represent specific variations of established chemical reactions previously described in the literature, but brought together in a new single scheme to produce the desired novel product in good yield and with no major safety concerns. This scheme allows a qualified investigator or contract laboratory to produce the inhibitor with standard laboratory facilities.

All intermediates were confirmed by HPLC and mass spectrometry (MS). The structure and purity of the final compound were confirmed by HPLC, MS and ^1^H-NMR. A Dionex HPLC system (Dionex, Sunnyvale, CA) equipped with a Dionex P680 pump and UVD170U ultraviolet detector was used for analytical and bioanalytical analyses of tissue extracts, equipped with a Phenomenex (Torrance, CA) Luna C18 column (250 × 2.0 mm; 5 μm) and guard column with a flow rate of 0.2 mL/min. The mobile phase consisted of 0.1 % (v/v) formic acid (HPLC grade; Fluka/Sigma-Aldrich, St. Louis, MO) in HPLC grade water as reagent A and either 100% acetonitrile (HPLC grade; Burdick & Jackson, Morristown, NJ) or 80% acetonitrile/0.08% formic acid/water as reagent B. UV absorption was monitored at four wavelengths (215, 230, 260 and 300 nm) with the 260 nm trace being the standard one used for quantification.

### Compound 1: 5-bromo-6-phenylpyridazin-3(*2H*)-one

The starting material compound **1 **was obtained from GL Synthesis Inc. (Worcester, MA) and manufactured in two steps using Friedel-Crafts alkylation of benzene with mucobromic acid to make 2,3-dibromo-4-phenylcrotonolactone, which upon treatment with hydrazine yields 5-bromo-6-phenylpyridazin-3(*2H*)-one **1 **[61-63].

### Compound 2: 4,6-dichloro-3-phenylpyridazine

Chlorination of compound **1 **with phosphorus oxychloride (10 eq, > 99%) yielded 4,6-dichloro-3-phenylpyridazine, or compound **2**. The reaction was done under reflux (90°C) for 2 hrs, with 5 M NaOH solution used to control the HCl formed during the course of reaction. After completion, the reaction mixture was cooled to ambient temperature and poured onto crushed ice. The mixture was neutralized with a 5 M NaOH solution to give a white suspension. The suspension was filtered on a medium frit sintered glass funnel to collect the solid. The filter cake was washed three times with deionized water and air dried on the filter to provide the compound **2 **in 99% gravimetric yields. ESI m/z (MeOH): 225.4 (MH^+^).

### Compound 3: 6-chloro-3-phenylpyridazin-4-ol

Compound **2 **and NaOH (1.85 eq,) were suspended in deionized water and heated under reflux (130°C) until a clear solution formed (~2 hrs). The reaction mixture was cooled in an ice water bath and acidified with HCl to pH = 1. The forming solid was filtered on medium frit sintered glass funnel and washed with 2 M Na_2_CO_3 _solution. The collected filtrate was again acidified with 2 M HCl to pH = 1, generating a white solid, which was collected by filtration on a medium frit sintered glass funnel, washed with deionized water and taken to dryness on the filter. The solid obtained was the desired product **3 **in 67% gravimetric yield. ESI m/z (MeOH): 207.03 (MH^+^).

### Compound 4: 3-phenyl-6-(4-(pyrimidin-2-yl)piperazin-1-yl)pyridazin-4-ol

Compound **3 **and 1-butanol were placed in a heavy wall pressure vessel (Chemglass, Vineland, NJ) and 1-(2-pyrimidyl)piperazine (4 eq) was added. The pressure vessel was closed and heated at 130°C for 26 hrs. The reaction mixture was cooled to ambient temperature, transferred to a round bottom flask and concentrated *in vacuo*. The residue was treated with deionized water and the precipitate was collected by filtration on a medium frit sintered glass funnel, the filter cake washed three times with deionized water, and dried over a filter funnel *in vacuo *to give the desired product **4 **in 98% gravimetric yield. ESI m/z (MeOH): 335.2 (MH^+^).

### Compound 5: 4-chloro-3-phenyl-6-(4-(pyrimidin-2-yl)piperazin-1-yl)pyridazine

Compound **4 **was suspended in phosphorus oxychloride (ReagentPlus Grade, > 99%). The reaction mixture was heated under reflux (90°C) for 2 hrs, cooled to ambient temperature and poured onto crushed ice. The mixture was neutralized with 5 M NaOH solution to give a white suspension. The precipitate was collected by filtration on a medium frit sintered glass funnel, the filter cake was washed with deionized water and dried *in vacuo *to give the product **5 **in 99% gravimetric yield. ESI m/z (MeOH): 353.3 (MH^+^).

### Compound 6 (MW01-2-069A-SRM): 3-phenyl-4-(pyridin-4-yl)-6-(4-pyrimidin-2-yl)piperazin-1-yl)pyridazine

Compound **5 **and pyridin-4-yl boronic acid (1.35 eq, Sigma-Aldrich, St. Louis, MO) were suspended in dimethoxyethane and water in a heavy wall pressure vessel (Chemglass, Vineland, NJ). The reaction mixture was purged with argon for 10 min. Tetrakis(triphenylphosphine)palladium (0.07eq, STREM Chemicals, Newburyport, MA) and sodium carbonate (3 eq) were added and the reaction mixture was heated at 110°C for 15 hrs. The reaction mixture was cooled to ambient temperature and filtered though a medium frit sintered glass funnel filled with Celite^® ^545. The filtrate was concentrated *in vacuo*, dissolved in ethyl acetate and washed with deionized water. The organic layer was dried with sodium sulfate, concentrated *in vacuo*, triturated with hexanes, and recrystallized from ethanol. The final product was obtained as light yellow crystals in 74% gravimetric yield. The final purity of the inhibitor was examined by reverse phase HPLC (> 99%) and the structure was confirmed by mass spectrometry (ESI) and ^1^H-NMR. ESI m/z (MeOH): 396.3 (MH^+^).^1^H-NMR (CDCl_3_): 8.57 (d, J = 5.7 Hz, 2H); 8.35 (d, J = 4.7 Hz, 2H); 7.34 (d, J = 6.7 Hz, 2H); 7.25 – 7.32 (m, 3H); 7.14 (d, J = 5.7 Hz, 2H); 6.89 (s, 1H); 6.56 (t, J = 4.7 Hz, 1H); 4.03 (t, J = 4.7 Hz, 4H); 3.87 (t, J = 4.7 Hz, 4H). HPLC (t_r_/purity): 18.97 min/> 99%. Melting point: 221.3 – 221.5°C.

### Synthesis of MW01-4-119SRM, *m*-pyridinyl analog of compound MW01-2-069A-SRM: 3-phenyl-4-(pyridin-3-yl)-6-(4-(pyrimidin-2-yl)piperazin-1-yl)pyridazine

Compound **5 **and pyridin-3-yl boronic acid (1.37 eq, Sigma-Aldrich, St. Louis, MO) were suspended in dioxane/water (4 mL/1 mL) in a 10 mL microwave glass vessel. The reaction mixture was purged with argon for 5 min. Tetrakis(triphenylphosphine)palladium (0.1eq, STREM Chemicals, Newburyport, MA) and sodium carbonate (3 eq) added. Microwave irradiation of 30 W was used, the temperature being ramped from ambient to 150°C. Once the set temperature of 150°C was reached, it was maintained for 20 min. The reaction was allowed to cool to ambient temperature, ethyl acetate added, and the reaction mixture filtered through a medium frit sintered glass funnel filled with Celite^® ^545 and dried over MgSO_4_. The solvent was removed and the residue was triturated several times with hexanes. Recrystallization from ethylacetate afforded the desired product in 64% gravimetric yield. The compound purity was examined by reverse phase HPLC and confirmed by mass spectrometry (ESI). ESI m/z (MeOH): 396.53 (MH^+^). HPLC (t_r_/purity): 19.99 min/> 98%.

### Synthesis of other compounds

Minozac, MW01-3-183WH, and MW01-6-189WH were synthesized, purified, and characterized as previously described [22].

### Aqueous solubility determination (log S)

For aqueous solubility (log S) determination, compound 069A was weighed on a Sartorius AG (Goettingen, Germany) analytical balance, and Milli-Q water was added to create an oversaturated solution (suspension). Sample tubes were stirred for 24 hrs at ambient temperature (t = 22°C). 1 mL of the samples were centrifuged in a microfuge at 16,000 × g for 10 min, and 20 μL was subjected to reverse-phase HPLC analysis to determine the concentration of the compound (c_aq_). The concentration of compound in the aqueous phase (c_aq_) was determined by peak detection at 254 nm at the appropriate retention time relative to a standard curve obtained from serial dilutions of the compound. The log S was calculated as common logarithm (log_10_) of c_aq_.

### Lipophilicity determination (log P)

The partition coefficient was determined by the shake-flask method [64-67]. The starting solution (0.3 mg/mL) of analyzed compound was prepared in presaturated 1-octanol (Sigma-Aldrich, St. Louis, MO). 1 mL of the octanol phase was agitated on a plate shaker with 10 mL of presaturated Milli-Q water for 2 hrs at 25°C. After partitioning, 1 mL of the aqueous phase was centrifuged in a microfuge for 10 min at 16,000 × g. 20 μL of the sample were analyzed with reversed-phase HPLC as described above to determine the concentration of the aqueous phase (c_aq_). The log P is calculated according to the formula: log P = log_10 _[(0.3 – c_aq_)/c_aq_].

### Protein kinase inhibitor activity

Concentration-dependent inhibition of protein kinase activity was done essentially as previously described [68]. Active p38α/SAPK2α enzyme was obtained from Millipore (Billerica, MA), and bovine myelin basic protein substrate was obtained from Sigma-Aldrich (St. Louis, MO). Enzyme activity assays were done in a final volume of 50 μl and each point was tested in duplicate. Assays were done by incubation with 0.33 mg/ml (18 μM) myelin basic protein, 100 μM ATP and γ-[^32^P]ATP (specific activity > 300 cpm/pmol) in assay buffer (50 mM HEPES, pH 7.5, 5 mM MgCl_2_, 150 mM KCl, 15 mM NaCl, 1 mM DTT). Reactions were initiated by addition of the active p38α kinase at a final concentration of 2 μg/ml, and incubated for 10 min at 30°C. Reactions were stopped by transfer of a 35 μl aliquot of the assay mixture onto P81 paper (Whatman, Clifton, NJ), washes with 75 mM phosphoric acid and 95% ethanol, and quantification by scintillation counting. When compounds were tested for kinase inhibitory activity, 10X stock solutions of compounds were prepared in DMSO or water, and control samples contained the same final concentration of solvent as the samples containing compound. Data are expressed as percent of the maximal enzyme activity, where enzyme activity in the absence of compound is taken as 100%. IC_50 _values were calculated by a nonlinear regression data analysis using Microsoft Excel. Selectivity against pathway or structurally related protein kinases was done using purified kinases and standard *in vitro *assays described previously [69,70], and included use of the Millipore (Billerica, MA) kinase screening service at 20 μM final concentration of 069A and 90 μM final concentration of ATP.

### Oral bioavailability and brain uptake analysis

Analysis of oral bioavailability (concentration of compound in the blood as a function of time after oral administration) and brain uptake was done by a modification of that previously described [22]. Briefly, 069A (2.5 mg/kg) was administered to C57Bl/6 mice by oral gavage in a 0.5% (w/v) carboxymethylcellulose suspension. At 5, 15, 30, 60, and 120 min after compound administration, blood was collected in heparinized tubes from anesthetized animals and plasma obtained by centrifugation. After perfusion, brains were harvested, homogenized in 0.1% formic acid and deproteinized with ice-cold acetonitrile. After centrifugation to remove precipitated protein, the brain homogenate supernatants were further diluted with 0.1% formic acid. The plasma samples were acidified by diluting with 0.1% formic acid (1:3, v:v). Solid phase extraction followed by HPLC analysis was used to quantify the amount of compound in the plasma and brain supernatants, with compound MW01-6-189WH used as an internal standard. Briefly, 30 mg cartridges (Oasis^® ^HLB, Waters) were conditioned with 1 mL of acetonitrile and equilibrated with 1 mL of water. Acidified samples were loaded onto the cartridge followed by a 1 mL wash with 5% acetonitrile. Compound was eluted from the cartridge using 100% acetonitrile. The eluate was evaporated to dryness, reconstituted in 60% acetonitrile/0.1% formic acid/water and analyzed by HPLC. Recovery of internal standard and compound 069a was approximately 70%.

### Liver toxicity screen

Screening for histological liver injury after chronic, therapeutic dose administration of compound was done as previously described [22,23]. Briefly, C57Bl/6 mice were administered by oral gavage either 069A (2.5 mg/kg) or solvent control (DMSO) in a 0.5% (w/v) carboxymethylcellulose suspension. Compound or vehicle administration was once daily for two weeks. Livers were then removed, fixed in 4% (v/v) paraformaldehyde, and paraffin-embedded for histology. To assess histological toxicity, 4 μm liver sections were stained with haematoxylin and eosin. Two independent observers blinded to the treatment groups performed microscopic assessment of the tissue for injury by using a semi-quantitative histological scoring system that considers architecture features (normal to extensive fibrosis), cellular features (normal to extensive edema and widespread necrosis), and degree of inflammatory infiltrate (normal to extensive infiltrate).

### Stability in human liver microsomes

Analysis of metabolic stability of compound 069A, Minaprine (Sigma-Aldrich), and Minozac [22] was tested *in vitro *by using commercially available human liver microsomes and an NADPH-regenerating system (BD Biosciences Discovery Labware; Woburn, MA), by the method previously described [71]. Briefly, triplicate reaction mixtures contained 0.1 M potassium phosphate buffer (pH 7.4) and a final concentration of the following components: 1 – 1.6 mg/ml total microsomal protein, 5 μM test compound, 1.3 mM NADPH, 3.3 mM MgCl_2_, 0.4 U/ml glucose-6-phosphate dehydrogenase, and 3.3 mM glucose-6-phosphate in a total volume of 300 μl. Mixtures were incubated at 37°C for 10 or 30 minutes. Reactions were terminated by addition of ice cold acetonitrile, centrifuged at 12,000 × g for 10 min to pellet precipitated microsomal protein, and the supernatant analyzed by HPLC to quantify the percentage of the initial amount of parent compound remaining after incubation. HPLC was performed as described above with 0.1% (v/v) formic acid in water as reagent A and acetonitrile with 0.1% (v/v) formic acid in water as reagent B. Peak quantification was done based upon absorption measurements at 260 nm relative to a standard curve obtained by serial dilutions of compounds. Control incubations revealed loss of compounds due to microsomal binding was less than 10%.

### *In vivo *efficacy in AD mouse model

The four-week intracerebroventricular (ICV) infusion of human oligomeric Aβ_1–42 _or Hepes/HDL vehicle into C57Bl/6 mice was done as previously described [22,23]. Mice were administered by oral gavage either 069A (2.5 mg/kg) or solvent control (DMSO) in a 0.5% (w/v) carboxymethylcellulose suspension. Compound administration began at day 21 after the start of Aβ infusion, and continued on a once daily administration schedule for 14 days. Beginning at day 50 after the start of Aβ infusion, the Y-maze test of spontaneous alternation was done once daily for 10 days to evaluate hippocampal-dependent spatial learning as described previously [23]. At day 60, mice were anesthetized, perfused, and sacrificed as previously described [23]. Hippocampal extract supernatants were prepared by dounce and sonication, followed by centrifugation as described previously [23]. Levels of the proinflammatory cytokines IL-1β and TNFα in hippocampal supernatants were measured by ELISA (Biosource International) per the manufacturer's instructions. S100B and synaptophysin levels were measured by ELISA as previously described [23].

### Statistical analyses of *in vivo *results

Experimental and control groups were compared using one-way ANOVA with Newman-Keuls post-hoc analysis using GraphPad Prism, version 4.00 statistical software. Significance was assumed when p < 0.05.

## Results and Discussion

### Development and characterization of a novel p38 MAPK inhibitor with potential use for CNS studies

**Figure 2:**
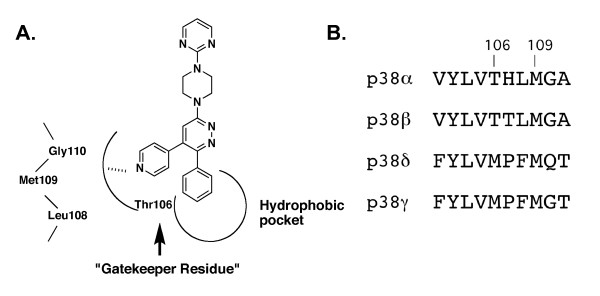
**Design of MW01-2-069A-SRM. A) **Pharmacophore model showing the potential for 069A to make selective interactions in the active site of p38α MAPK. The pyridine ring nitrogen has the potential to make the critical interaction with the hydrogen of the amide bond formed between Met109 and Gly110. This interaction and the potential to occupy the nearby hydrophobic pocket are important interactions for p38α MAPK selective inhibitors. The "gatekeeper residue" in p38α and p38β is Thr106. Its small size compared to the larger Met in p38δ and p38γ isoforms allows bulkier groups in the compound to access the pocket, thereby providing isoform selectivity and potential affinity. **B) **Amino acid sequence alignment of p38 MAPK isoforms in the region containing key amino acids implicated in selective kinase-inhibitor interactions.

**Figure 3:**
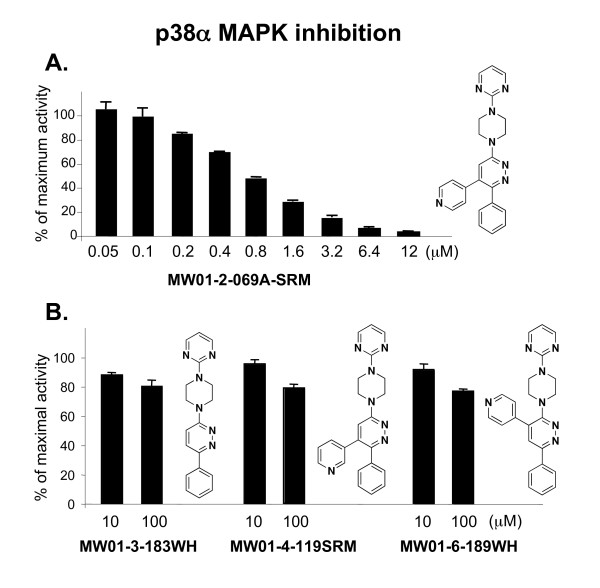
**Inhibition of p38α MAPK enzyme activity and validation of design for selective interactions. A) **MW01-2-069A-SRM inhibits p38α MAPK enzyme activity in a concentration-dependent manner. **B) **In contrast, the 069A analogs are > 100-fold less active. These include the starting scaffold (MW01-3-183WH), an analog (MW01-4-119SRM) with a different orientation of the pyridine ring nitrogen such that potential H-bond interactions are minimized, and an analog (MW01-6-189WH) with the pyridine ring at a different position on the scaffold. The *in vitro *phosphorylation of the standard protein substrate, myelin basic protein, by purified p38α MAPK was measured as described in Methods, in the absence or presence of increasing concentrations of compound. Data are expressed as percent of the maximal enzyme activity, where enzyme activity in the presence of solvent only (absence of inhibitor) is taken as 100%. Data are a representative example of five experiments.

The synthetic scheme (Fig. 1) and design strategy (Fig. 2) for the p38 MAPK inhibitor 069A were based on a chemical diversification of the inactive 3-phenyl-6-(4-(pyrimidin-2-yl)piperazin-1-yl)pyridazine scaffold (MW01-3-183WH; Fig. 3), used in previous development of CNS-penetrant, orally bioavailable, non-toxic, experimental therapeutics [22,23]. Computational modeling predicted that the scaffold should fit into the p38α MAPK structure, with the phenyl ring occupying a hydrophobic pocket in the kinase (Fig. 2A). To create the potential for further interaction with the p38 MAPK active site, we introduced into the scaffold a pyridinyl pharmacophore found in a variety of p38 MAPK inhibitors [72-74]. The nitrogen of the pyridine ring is potentially able to make a critical H-bond interaction with the amide bond formed between Met109 and Gly110 of p38α MAPK. Therefore, we incorporated this feature into the design of 069A.

The activity of 069A as a p38α MAPK inhibitor was tested in an *in vitro *protein kinase assay over a wide concentration range (0.05 – 12 μM). As shown in Fig. 3A, 069A inhibits p38α MAPK activity in a concentration-dependent manner with an estimated IC_50 _of 0.8 μM. As shown in Fig. 3B, the starting scaffold (compound MW01-3-183WH) lacks inhibitory activity, consistent with the model in Fig. 2. Therefore, introduction of the pyridinyl pharmacophore adjacent to the phenyl ring of the inactive scaffold to generate 069A results in a greater than 100-fold increase in inhibitory activity.

To further validate the design approach and specificity of the inhibitor-kinase interaction, we examined in more detail the finding that introduction of the pyridinyl pharmacophore into the inactive scaffold generated kinase inhibitory activity. The attainment of p38 MAPK inhibitory activity is dependent on the molecular context of the pyridinyl pharmacophore placement. For example, we synthesized an analog, called MW01-4-119SRM, to test the activity importance of the potential H-bond interaction with the Met109 peptide backbone of the kinase. We used the same synthetic scheme as used for 069A, but a different pyridinylboronic acid (Fig. 1, step e), allowing a different structural orientation of the nitrogen in the pyridine ring. If the proposed interaction (Fig. 2) involves such an H-bond, one would anticipate that activity would be compromised due to distance constraints and altered electronegativity. As shown in Fig. 3B, there was a major loss of activity back toward that seen with the scaffold alone. The remarkable gain in activity with the introduction of the pyridinyl pharmacophore into the scaffold to generate 069A is also dependent on the location of the pyridine on the scaffold. For example, placement of the pyridine ring at a different location on the pyridazine ring, such as in compound MW01-6-189WH (Fig. 3B), does not generate MAPK inhibitory activity (Fig. 3B). Therefore, compounds with identical compositions to 069A, but different structures, are not able to replicate the kinase inhibitory activity of 069A.

Another feature of the model in Fig. 2 relates to the "gatekeeper residue" characteristic of many protein kinases. In p38α and p38β, the gatekeeper residue is Thr106, whereas a larger Met residue is found in the analogous position in p38δ and p38γ (Fig. 2B). The smaller Thr residue allows bulkier groups, such as the phenyl at position 6 of the pyridazine ring in 069A, to access the hydrophobic pocket found in the kinase active site. Based on structure parameters, therefore, we would predict that 069A should be a selective inhibitor of p38α and p38β, but not be an inhibitor of the p38δ and p38γ isoforms. This selectivity was validated as part of an *in vitro *protein kinase screen done by Millipore (Billerica, MA); at 20 μM, 069A showed complete inhibition of p38α enzyme activity, partial inhibition of p38β, and no inhibition of p38δ or p38γ (Table 1). In addition, there was little or no inhibition by 069A of 44 other purified protein kinases (Table 1). Clearly, the pyridine pharmacophore must be introduced adjacent to the phenyl group in the molecular context of the scaffold, as found in compound 069A, to generate a p38α MAPK-selective inhibitor.

**Table 1:** MW01-2-069A-SRM selectivity in standard kinase profiling screen

**Kinase**	**% kinase activity**	**Kinase**	**% kinase activity**	**Kinase**	**% kinase activity**	**Kinase**	**% kinase activity**
ASK1	107	IRAK4	112	MLK1	94	PKBβ	91
CDK1/cyclinB	94	JAK2	108	MSK1	94	PKBγ	100
CDK2/cyclinA	94	JNK1α 1	90	MSK2	96	PRAK	86
CDK5/p25	90	Lyn	94	p70S6K	111	Pyk2	83
CK2	96	ERK1	90	**p38α **	**-2**	ROCK-I	89
FAK	96	ERK2	99	**p38β **	**30**	RSK1	100
Fyn	98	MAPKAP-K2	97	p38δ	101	Syk	96
GSK3β	74	MAPKAP-K3	94	p38γ	95	TAK1	97
IKKα	95	MEK1	104	PAK2	116	TAO1	94
IKKβ	104	MKK4	117	PDK1	101	TAO2	92
IRAK1	110	MKK7β	103	PKBα	76	TBK1	95

The ability of 069A (20 μM final concentration of compound; 90 μM final concentration of ATP) to suppress the activity of purified kinases was determined in a Millipore/Upstate kinase profiling assay as described by the manufacturer. Kinase activity in the absence of inhibitor is taken as 100%.

Approved drugs and experimental therapeutics that are protein kinase inhibitors generally exhibit inhibition constants ≤ 1 μM and perturb intracellular signal cascades. This apparent affinity of efficacious kinase inhibitors is consistent with their ability to compete with the endogenous substrates (ATP and physiological protein substrates), for which kinases generally have Km values in the 1–20 μM range. This requires that an inhibitor have comparable or better affinity for the kinase than its physiological substrates for efficacy. The affinity and selectivity of 069A for inhibition of p38 MAPK activity justified further biological characterization of this compound. However, in order to interpret the *in vivo *biological effects of such an inhibitor, there must be some indication that the compound is bioavailable, stable and non-toxic.

### Molecular properties and bioavailability of the p38α MAPK inhibitor

Molecular properties are physical features of small molecules that generally define what makes a chemical drug-like and likely to be taken up into the bloodstream (bioavailable) because they are associated with the ability to exist in aqueous biological milieu yet able to reversibly penetrate biological membranes. A compound's molecular properties, such as lipophilicity and aqueous solubility, also contribute to whether or not the drug is rapidly metabolized by liver enzymes after being consumed by mouth, and are a major determinant of whether the compound is one of the approximately 2% of small molecule drugs that have blood-brain barrier penetrance [60]. The *de novo *compound discovery platform [22,23,29] used to develop 069A uses considerations of multiple molecular properties at both the design and synthetic compound characterization stages. While the physical properties associated with bioavailability and brain penetrance are often estimated by commercially available computational algorithms as part of compound design, they are only approximations to assist in design and often deviate significantly from experimentally determined values [29]. Therefore, we determined the experimental values for lipophilicity and aqueous solubility of 069A using the protocols described in Methods. The experimentally determined lipophilicity of 069A yields a log P value of 3.18. This value is within the range of computed and experimentally determined values found for orally bioavailable, CNS-penetrant drugs [29]. Similarly, the experimentally determined aqueous solubility of 069A is 9.54 μg/mL, which translates to a log S value of -2.02, and is a value within the desired range [29].

**Figure 4:**
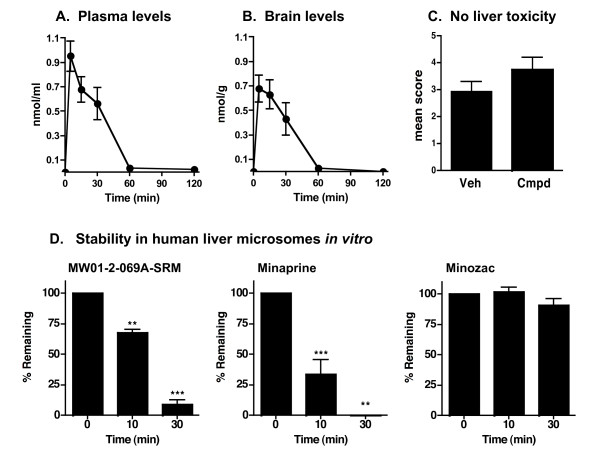
**Oral bioavailability, brain uptake, safety and metabolic stability of 069A**. Compound bioavailability **(A and B) **was determined by administration of 069A (2.5 mg/kg) by oral gavage to C57Bl/6 mice, processing of blood and brain at different times after administration, and measuring compound levels in plasma and brain homogenate extracts by HPLC as described in Methods. Compound 069A rapidly appears in plasma (**A**) and brain (**B**) within 5 min, and then slowly declines to basal levels by 60 min. Data are the mean ± SEM from 5 mice at each time point. Lack of idiopathic tissue injury **(C) **after chronic administration of compound was assessed by treating C57Bl/6 mice (5 mice per group) with either vehicle (Veh) or 069A (Cmpd; 2.5 mg/kg) by oral gavage once daily for two weeks. Histological liver injury was determined by a scoring system as described in Methods. There is no significant liver toxicity after chronic two-week administration of compound compared to vehicle, the same dose and paradigm used in efficacy testing. To screen for potential metabolic instability (**D**), compound 069A, the clinical CNS drug Minaprine, or the drug Minozac were incubated with human liver microsomes, and the amount of compound remaining was analyzed by HPLC as described in Methods. Significantly different from time 0 (**p < 0.01; ***p < 0.001).

The design and experimentally determined properties of the unformulated 069A suggest its potential to be orally bioavailable, CNS-penetrant, stable and non-toxic based on previous findings with this scaffold [22,23]. Experimental analyses (Fig. 4) confirmed this assumption. Specifically, 069A has acceptable oral bioavailability properties for use with *in vivo *studies. The compound is readily detected in the plasma within the earliest possible time point (5 min) analyzed after oral administration to mice, with bulk clearance from plasma within 60 min after oral administration (Fig. 4A). A similar pattern of time-dependent change in concentration is seen in the brain homogenates (Fig. 4B) prepared after perfusion of animals to remove any compound present in adventitiously associated blood. The peak brain concentration of 069A is seen at 5–15 min after oral administration, with bulk compound clearance by 60 min. Compound 069A did not induce any major untoward tissue injury, as assessed by histologic screening for idiopathic liver injury. Specifically, histological assessment of liver tissue showed that oral administration of 069A at 2.5 mg/kg daily for 2 weeks did not induce any histological indices of liver tissue injury compared with mice treated with the vehicle (Fig. 4C), and no adverse clinical effects of 069A administration were observed during the course of treatment. Finally, 069A also showed acceptable metabolic stability in human liver microsomes, with ~70% of the compound remaining after 10 min incubation with microsomes (Fig. 4D). Although 069A is not as metabolically stable as Minozac, it is more stable than the structurally related, clinical CNS drug Minaprine (Fig. 4D).

These data document that 069A has lipophilicity and solubility properties that fall within the working range of values for these molecular properties in CNS drugs [29], and that the compound is sufficiently bioavailable, metabolically stable, non-toxic, and brain-penetrant for *in vivo *efficacy testing in CNS pathology models.

### *In vivo *efficacy in AD animal model

**Figure 5:**
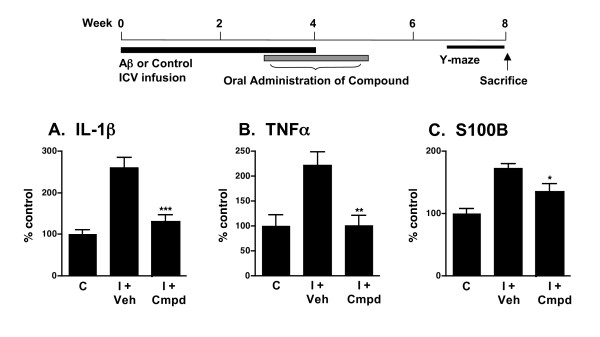
**Compound 069A suppresses proinflammatory cytokine up-regulation in AD mouse model**. A schematic of the experimental paradigm is shown. Treatment with 069A resulted in significant suppression of the Aβ-induced increase in the hippocampal levels of the proinflammatory cytokines IL-1β **(A)**, TNFα **(B) **and S100B **(C)**. Mice were infused ICV with either the diluent control (C) or the injurious oligomeric form of human Aβ_1–42 _(I) for 4 weeks. Starting at 3 weeks after the start of Aβ infusion, mice were administered by daily oral gavage either 069A (2.5 mg/kg; I + Cmpd) or vehicle (I + Veh) for two weeks. Spontaneous alternation of mice was measured for 10 days in the Y-maze, a hippocampus-dependent spatial learning task, beginning at day 50 after the start of Aβ infusion. Mice were sacrificed on day 60, and hippocampal extracts analyzed by ELISA. Data are means ± SEM of n = 5 mice per group. Significantly different from Aβ-injured: *p < 0.05, **p < 0.01, ***p < 0.001.

The *in vivo *efficacy of orally administered 069A was tested in a mouse model of AD-relevant pathophysiology that involves ICV infusion of oligomeric Aβ_1–42 _[22,23]. Animal models using ICV infusion of Aβ have good phenotypic penetrance of pathophysiology endpoints, including proinflammatory cytokine up-regulation, synaptic dysfunction, and hippocampal-dependent behavioral deficits, and have been used to identify compounds now in clinical trials [22,75]. The experimental design and treatment paradigm are shown diagrammatically in Fig. 5. The dose of 069A chosen to test was based on previous *in vivo *success with other suppressors of proinflammatory cytokine up-regulated production based on the same scaffold [22,23].

**Figure 6:**
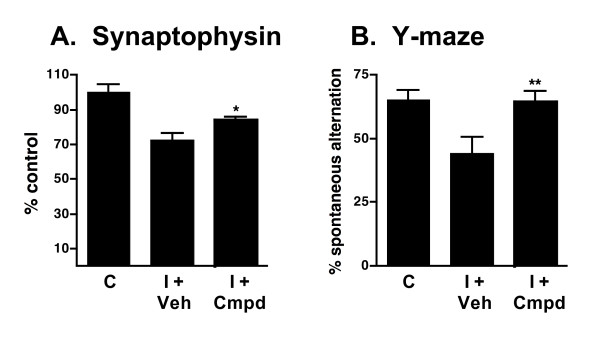
**Compound 069A attenuates synaptic protein loss and behavioral deficits in AD mouse model**. Treatments, behavioral testing, and preparation of hippocampal extracts were done as described in Figure 5. Treatment with 069A significantly attenuated Aβ-induced loss of synaptophysin **(A) **and the behavioral deficit in the Y-maze **(B)**. Data are means ± SEM of n = 5–12 mice per group. Significantly different from Aβ-injured: *p < 0.05, **p < 0.01.

As shown in Fig. 5, Aβ induces an increase in the levels of the proinflammatory cytokines IL-1β, TNFα and S100B in hippocampal extracts. Once-daily oral administration of a low dose (2.5 mg/kg) of 069A for 2 weeks, using a translational medicine paradigm of therapy after start of injury (21 days after the start of Aβ infusion), significantly reduced the overproduction of IL-1β (Fig. 5A), TNFα (Fig. 5B), and S100B (Fig. 5C) back toward basal. As shown in Fig. 6, Aβ exposure decreases the level of the presynaptic marker protein synaptophysin in hippocampal extracts, and induces a deficit in the Y-maze test of hippocampal-dependent spatial behavior. Oral administration of 069A attenuates this loss of synaptophysin (Fig. 6A) and ameliorates the Y-maze behavioral deficit (Fig. 6B). These results demonstrate that an orally active, brain-penetrant, small molecule inhibitor of p38α MAPK is efficacious in an AD-relevant animal model. Our findings indicate the importance of this protein kinase mediated pathway in AD-relevant overproduction of proinflammatory cytokines and its linkage to neuroinflammation-related neuronal dysfunction and behavioral deficits.

### p38α MAPK as a potential CNS therapeutic target

Protein kinases constitute an important class of druggable protein targets [76-78] and the multi-kinase inhibitors Gleevec (imatinib mesylate; Novartis), Nexavar (sorafenib; Bayer, Onyx), and Sutent (sunitinib malate; Pfizer) for treatment of various types of cancers are evidence of the potential for kinases to be therapeutic targets. These prior successes in cancer therapeutic development suggest the still untapped potential with respect to a multitude of other disease indications, such as CNS disorders. The novel compound development and *in vivo *biology results presented here add to an accumulating body of knowledge supporting protein kinases as being potential therapeutic targets relevant for new CNS disease therapeutics, if small molecules with appropriate molecular properties and activity can be developed.

Bioavailability is required but is not sufficient to make a chemical into a drug. *In vivo *efficacy also requires selective inhibitory activity at the level of the target protein kinase. The design of 069A exploited structural features previously identified [73] as being important for selectivity among MAPK isozymes. The validity of these design assumptions was confirmed by the synthesis and testing of analogs less likely to exploit the key inhibitor-kinase interactions. Therefore, the use of the Minozac scaffold to exploit its potential for bioavailability and brain penetrance, and chemical diversifications that allowed use of interactions selective for p38α MAPK and p38β MAPK over the other p38 MAPK isozymes, appear to be the molecular basis of the *in vivo *efficacy of compound 069A for brain dysfunctions.

The study summarized here does not unequivocally address if the improved neurologic outcome is due solely to the *in vivo *inhibition of glia p38 MAPK and proinflammatory cytokine production. For example, *in vitro *co-culture studies have shown [79] that inhibition of neuronal p38 MAPK prevented decreases in synaptophysin levels correlated with neuronal tau phosphorylation. Therefore, it is possible that inhibition of neuronal p38 MAPK activity by 069A may have contributed to some of the *in vivo *synaptic changes seen in the animal model studies reported here. However, this is not a major concern for therapeutic development, as the individual effects on glia proinflammatory cytokine production and neuronal signaling would both contribute to the overall positive neurologic effects observed. In fact, such multi-functional effects in the CNS due to p38α MAPK inhibition in different cell types might be advantageous for use in a variety of neurodegenerative disorders.

The *in vivo *behavior and functional effects of 069A allowed its use to test hypotheses in this study and the results provide a foundation for future drug development efforts. Targeting a protein kinase that can modulate gene transcription and translation allows the possibility that biological effects of the drug can continue past the time point when the bulk drug concentration in the brain is back to basal. In other words, the drug pharmacodynamics (what the drug does to the body) could exceed its pharmacokinetics (what the body does to the drug) with the potential outcome being a disease-modifying therapeutic. Clearly, these are important issues to be addressed in future drug development investigations.

## Conclusion

The studies summarized here have several important implications. First, the data provide *in vivo *evidence supporting the hypothesis that the gene-regulating, serine/threonine protein kinase p38α MAPK is a potential therapeutic target for CNS disorders where elevated levels of proinflammatory cytokines have been implicated as a component of disease progression. Second, the results presented here and previously [22,23] demonstrate that distinct signal transduction cascades can be modulated by small molecules to achieve the same *in vivo *outcome, attenuation of up-regulated brain proinflammatory cytokine production with resultant improvement in neuropathology. Third, the methods and approach described here demonstrate that novel tools for *in vivo *CNS research can be readily developed by rational variations of existing drug scaffolds to produce analog molecules with the desired *in vivo *properties. The novel p38α MAPK inhibitor 069A provides not only an important research tool for addressing hypotheses about the role of p38 MAPK in a variety of CNS disorders, but also represents a potential foundation for future campaigns to develop neurodegenerative disease-modifying therapies focused on this key gene-regulating protein kinase.

## Abbreviations

069A-  MW01-2-069A-SRM.

Aβ- Amyloid-beta 1–42.

AD- Alzheimer's disease.

CNS- Central nervous system.

ICV- Intracerebroventricular.

IL-1β- Interleukin-1β.

MAPK- Mitogen-activated protein kinase.

TNFα- Tumor necrosis factor α.

## Competing interests

DMW and LVE are principal investigators on NIH and foundation grant funding that has development of novel CNS therapeutics as the goal. Northwestern University's technology transfer office has filed patent applications for novel compounds that include the ones described here.

## Authors' contributions

LM assisted with compound design and the synthetic scheme, did molecular property characterizations, and performed the protein kinase assays. HRR carried out the *in vivo *experiments. SMR performed chemical syntheses and analytical chemistry characterizations. WH assisted with synthetic scheme development and performed chemical syntheses. JMC participated in the efficacy experiments. LKM performed the structure-assisted design. LWC did the *in vitro *metabolic stability assays. LVE and DMW conceived of the project and study, participated in its execution and coordination, and drafted the manuscript with the assistance of the other authors, who read and approved the resultant manuscript.
